# New Advances in the Study of Bone Tumors: A Lesson From the 3D Environment

**DOI:** 10.3389/fphys.2019.00814

**Published:** 2019-06-26

**Authors:** Margherita Cortini, Nicola Baldini, Sofia Avnet

**Affiliations:** ^1^Orthopaedic Pathophysiology and Regenerative Medicine Unit, IRCCS Istituto Ortopedico Rizzoli, Bologna, Italy; ^2^Department of Biomedical and Neuromotor Sciences, University of Bologna, Bologna, Italy

**Keywords:** 3D culture, sarcoma, bone metastasis, tumor niche, microenvironment

## Abstract

Bone primary tumors, such as osteosarcoma, are highly aggressive pediatric tumors that in 30% of the cases develop lung metastasis and are characterized by poor prognosis. Bone is also the third most common metastatic site in patients with advanced cancer and once tumor cells become homed to the skeleton, the disease is usually considered incurable, and treatment is only palliative. Bone sarcoma and bone metastasis share the same tissue microenvironment and niches. 3D cultures represent a new promising approach for the study of interactions between tumor cells and other cellular or acellular components of the tumor microenvironment (i.e., fibroblasts, mesenchymal stem cells, bone ECM). Indeed, 3D models can mimic physiological interactions that are crucial to modulate response to soluble paracrine factors, tumor drug resistance and aggressiveness and, in all, these innovative models might be able of bypassing the use of animal-based preclinical cancer models. To date, both static and dynamic 3D cell culture models have been shown to be particularly suited for screening of anticancer agents and might provide accurate information, translating *in vitro* cell cultures into precision medicine. In this mini-review, we will summarize the current state-of-the-art in the field of bone tumors, both primary and metastatic, illustrating the different methods and techniques employed to realize 3D cell culture systems and new results achieved in a field that paves the way toward personalized medicine.

## Introduction

Cancer is a complex disease that thrives in a heterogeneous and adaptive tumor microenvironment (Park et al., 2014). Bone sarcomas and bone metastasis (BM) share the same environment and the niche, where tumor cells can seed and proliferate. Osteosarcoma (OS), chondrosarcoma (CS), and ewing sarcoma (ES) are the most common malignant primary bone tumors, accounting for 70% of all such malignancies. Despite the advent of chemotherapy has widely improved patient survival, sarcomas are still considered deadly and, in a high percentage of cases, incurable diseases (Lewis, 2009). Similarly, BM form when carcinoma cells have homed to the skeleton and, at this stage, the disease is usually considered incurable, treatment with current modalities is only palliative and often associated to uncomfortable side effects (Fornetti et al., 2018).

Bone sarcomas are a disease of mesenchymal origin; they originate in the bone, where the mesenchymal stem cells (MSC) are both ontogenic progenitor tumor cells (Mohseny et al., 2009; Lye et al., 2016) and stromal cells that participates to tumor development (Xiao et al., 2013; Cortini et al., 2017). In the bone, the tumor-supporting stroma is formed by osteoblasts (the bone forming cells deriving from MSC), osteoclasts (the bone resorbing cells), endothelial and immune cells, and MSC. Osteoclasts adhere to bone surface and the spectrum of factors involved in their activation may depend on tumor type. As an example, osteoclasts can be metabolically fueled directly by tumor cells (Lemma et al., 2016, 2017) or also stimulated by tumor-induced osteoblasts (Sousa and Clezardin, 2018). In OS, the presence of osteoclasts in the tumor microenvironment may foster the osteoblastic behavior of tumor cells and increase their aggressiveness (Costa-Rodrigues et al., 2011) and is considered a bad prognostic factor (Salerno et al., 2008). Similarly, in BM, the pathogenic process forms when the delicate balance between bone deposition and resorption is disrupted (Alfranca et al., 2015).

Given the complexity and heterogeneity of bone tumors, the therapeutic strategies aimed at their eradication has exhibited a consistent slow-down to respect to many other carcinomas. Clearly, a better understanding of bone cancer oncogenesis is warranted to overcome drug resistance and improve low survival rate. A number of obstacles impede the study of bone cancers with the current means. These include the physical difficulty of manipulating bone as a tissue, the rarity of the tumors for sarcoma, the difficulties of obtaining tumor tissue fragments from human patients for BM, and the limited number of models that effectively mimic human disease. For all these reasons, the need for new cell models for bone cancers is becoming crucial.

In this review, we focused on the cellular models that are currently available for the study of BM or sarcomas. Such models have long been restricted to the two dimensions (2D) of dishes – an obvious obstacle to investigating structure and organization in cultured cells. However, a variety of 3D cell culture methods have recently emerged and are changing the way that multicellular systems are modeled.

## Advances in 2D Systems

**FIGURE 1:**
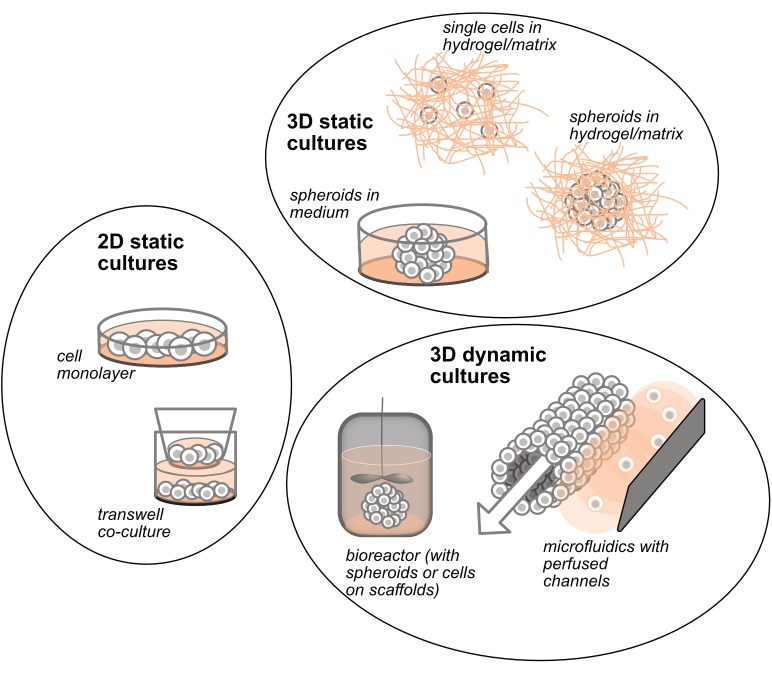
Schematic representation of the different and most used 2D and 3D *in vitro* culture systems.

For decades, monolayer cultures have been the leading light in wet biology; Harrison (1907) developed the first cell culture from a nerve fiber in 1907 and demonstrated that tissue specimens could live out of the body for as long as a 4 weeks time. Since then, 2D monolayers have been worldwide extended and the culture technique substantially improved. Nowadays, monolayer cultures have been upgraded for the study of single or multiple populations. Co-seeding, transwell membranes and conditioned culture medium are examples of how cells can be easily handled. Cancer cells can be treated with conditioned medium of other cells that play a consistent role in tumor growth (i.e., fibroblasts or MSC) (Sasser et al., 2007; Iser et al., 2016). Transwell allows the culturing of two cell types seeded in separate compartments (Figure 1; Chiovaro et al., 2015; Avnet et al., 2017). Lastly, co-seeding two different cell population in the same compartment is also possible, but discrimination of the studied effect on one of the two populations requires the physical separation of the cells that is expensive and not always possible (Molloy et al., 2009).

Despite this, differences in cell morphology, migration, polarization, interaction with the ECM but also cell metabolism and regional genotypic and phenotypic changes (Dhiman et al., 2005; Chitcholtan et al., 2013; Russell et al., 2017), or chemoresistance (Colella et al., 2018) are often far from being confirmed in 3D models. Altogether, these difference are surely due to the lack of spatial relationships and adequate culture conditions. Among the others, one example is that cells respond differently to hard substrates like lab plastic than they do on softer ones that resemble the ECM (Engler et al., 2006).

## The Upgrade to the *In Vitro* Third Dimension

Three dimensional architecture is one of the main issues at the basis of tissue and organ formation; this level of complexity starts during embryonic development and further enhances with cell-to-cell contacts that pose the basis to intracellular functions (Fitzgerald et al., 2015). Furthermore, cells are surrounded by an ECM that crucially determines cell differentiation, proliferation, and homeostasis (Kinney et al., 2014). An ideal 3D culture model should thus properly mimic not only oncogenesis, and maintenance of tumor cell growth, but also imitate the interactions between cells intermingled within the ECM. To date, with this aim, several technologies have been developed and explored: static 3D cultures include the seeding of cells in spheroid-like structure without the extracellular matrix, and the seeding of cells in matrices or scaffolds, made of natural or synthetic biomaterials (Figure 1); dynamic 3D cultures include either spheroids or scaffolds cultured in bioreactors, and the seeding of cells in microfluidic perfused devices (Figure 1).

### Spheroids

One of the pioneer studies that has opened the field to 3D cultures is the work by Sutherland et al. (1971); they were among the first to observe that lung cells grown in suspension would form spheroids that develop with an outer zone containing proliferating cells, a poorly nourished and oxygenated intermediate zone containing few cells in mitosis, and a central zone of necrosis, a typical feature of physiological tumor masses. Forced-floating spheroids are the simplest method to generate spheroids: cells are prevented to attach to the well bottom, resulting in floating aggregates and cell-cell contacts. The hanging-drop method is the most widely used and is a static technology (Kelm et al., 2003). Adversely, rotating cell culture bioreactor, spinner flasks or stirred-tank cultures (Santo et al., 2016) force homogenous spheroid formation by continuous agitation (Breslin and O’Driscoll, 2013). Also in this case, spheroids can be formed by a single cell type or can mimic interactions between multiple cells, such as tumor and stromal cells (Ishiguro et al., 2017). These culture systems are highly reproducible and have low production costs; already in 1971 it was clear that spheroids had the potential to be used for drug screening or to test radiation therapies (Sutherland et al., 1971). Despite the advantages, not all cell lines form spheroids and some form only unpredictable cell aggregates.

### Matrices and Scaffolds

As for spheroids, cells seeded into matrices or on scaffolds can be cultured either in static or dynamic cultures using rotating cell culture bioreactors.

A hydrogel-based matrix is a network of physically or chemically cross-linked polymer molecules of hydrophilic nature that allows to retain large amounts of water (Ahmed, 2015) and provide a 3D biomimetic environment supporting cell proliferation and differentiation (Peck and Wang, 2013). The major advantage of hydrogels is their customization according to the specific features of the ECM. As an example, hydrogels can be designed to shrink or swell based on the environmental stimuli that they receive (Ahmed, 2015), and can be easily enriched with specific cell adhesion ligands to mimic soft tissues. Hydrogels are of synthetic or natural origin (Li and Kumacheva, 2018), and are mainly based on matrigel, collagen or fibrin. Matrigel derives from a mouse sarcoma and has the most heterogeneous composition. The chief components are structural proteins such as laminin, nidogen, collagen, and heparan sulfate proteoglycans. Matrigel polymerization depends on temperature. Collagen-based hydrogels rely also on pH and plays a crucial role in cancer progression and is the most common protein of mammalian ECM. However, the pH-dependency makes collagen-based hydrogels unsuitable for the study of the effects of tumor acidosis, a feature that is crucial for the development of bone cancer (Cortini et al., 2017; Avnet et al., 2019), or of cancer-induced bone pain (Yoneda et al., 2015; Di Pompo et al., 2017).

Traditionally described as tools made of polymeric biomaterials, 3D scaffolds have the advantage to provide recapitulation of the ECM by providing, like hydrogels, attachment sites and interstitial space for the cells that can grow and proliferate, forming 3D structures (Breslin and O’Driscoll, 2013). Scaffold stiffness can be tuned to influence cell adhesion, proliferation and activation (Keogh et al., 2010). Materials used for scaffold fabrication must be biocompatible and must induce molecular biorecognition from cells (Carletti et al., 2011). ECM-mimicking biomaterials are made of collagen, hyaluronan, matrigel, elastin, laminin-rich-extracellular matrix, and also alginate, chitosan, silk and are considered as the most biocompatible. Synthetic biomaterials include polyethylen glycol, hyaluronic acid-PEG, polyvinyl alcohol, polycaprolactone, or two-phase systems such as polyethylene glycol-dextran. A number of biomaterials, such as ceramics, can fall in the natural or synthetic category (Thakuri et al., 2018).

### Microfluidic Devices

Recent advancements in tissue engineering have led to the development of living multicellular microculture systems, which are maintained in controllable microenvironments and function with organ level complexity [for an extensive review see Huh et al. (2011)]. The applications of these “on-chip” technologies are becoming increasingly popular for cancer studies (Sontheimer-Phelps et al., 2019). Continuous perfusion of media through the microfluidic network is the major innovation in these systems (Chung et al., 2017) since it mimics blood flow and enables exchange of nutrients, oxygen and metabolites with the blood tissue that are crucial for modeling living cancer tissues. Invading cells detaching from a solid tumor are exposed to the novel microenvironment of the circulatory system. Depending on the size of the vessel, the blood flow velocity can reach 0.03–40 cm/s, with arterial hemodynamic shear-force of 4.0–30.0 dyn/cm^2^ and venous shear-force of 0.5–4.0 dyn/cm^2^ (McCarty et al., 2016). Therefore, tumor cells must promptly adapt from static growth to fluid shear stress (Mitchell and King, 2013; Phillips et al., 2014), a condition that is far from being taken into account on static cultures. Until few years ago, microplates supported only 2D environments (Wu et al., 2010). More recently, the third dimension has been introduced to support 3D aggregates (Toh et al., 2007; van Duinen et al., 2015; Lanz et al., 2017). Finally, microfluidics has allowed the design and the development of self-organized organ-like cell aggregates that originate from multipotent stem cells, the organoids, and has opened a whole new level of biomimicry to be achieved (Yu et al., 2019). Representative examples are the blood brain barriers, the 3D neuronal networks, the kidney, liver or the intact gut epithelium or, when mentioning cancer tissues, glioma, breast cancer or sarcoma models (Sontheimer-Phelps et al., 2019).

This technology has the power to add multiple cell lines in the same chip. As an example, it is possible to mimic the tumor-endothelial cells interaction that is fundamental for the metastasization process, including angiogenesis, intravasation and cancer cell colonization (Zervantonakis et al., 2012). Likewise, microfluidics have been thoroughly studied to better recapitulate the cancer cell-immune cell interactions, with the ultimate aim of increasing knowledge on cancer immunotherapies (Boussommier-Calleja et al., 2016).

Finally, formation of 3D spheroids by using hanging drops have been combined to microfluidic platforms for drug testing or chemoresponses assays (Marimuthu et al., 2018). The next big challenge is the full validation of these models and subsequently the implementation in drug development pipelines of the pharmaceutical industry and ultimately in personalized medicine applications.

## Studying the Pathogenesis of Tumour Niche in 3D *In Vitro* Systems of Bone Cancers

Many papers have discussed the importance of switching from 2D to 3D cultures in a number of tumor cell lines (Praharaj et al., 2018), including bone sarcoma (De Luca et al., 2018). Novel models have now been acquainted also for bone cancers, and for tumor-related bone microenvironment (Table 1), and that include 3D tumor-resembling structure endothelial or fibroblastic cells in order to develop antiangiogenic therapies and to better understand vasculature expansion (Lee et al., 2006; Reddy et al., 2008; de Nigris et al., 2013). 3D OS cells have been combined with 2D endothelial HUVEC cells to form a well-organized network, including tubule-like structures that infiltrated the tumor spheroids, like new vessels *in vivo*. In this model, HUVEC proliferation and expression of angiogenesis-associated genes was possible induced by VEGF secretion from quiescent OS cells, embedded in matrix at the center of the spheroid, and stressed by the hypoxic core (Chaddad et al., 2017). The vasculature also seemed to direct the reactivation of dormant disseminated tumor cells. Targeting the vascular niches in such early steps of BM delays or even prevents the metastatic relapse (Kusumbe, 2016). Likewise, a functional tri-culture has been developed for studying metastatic breast cancer that has spread to the bone. This includes a stable vascular networks within a 3D native bone matrix cultured on a microfluidic chip; this niche-on-a-chip is characterized by controlled flow velocities, shear stresses, and oxygen gradients. Interestingly, MSC, which have undergone phenotypical transition toward perivascular cell lineages, support the formation of capillary-like structures lining the vascular lumen (Marturano-Kruik et al., 2018). MSC are associated with the tumor microenviroment since they are recruited by tumor cells from the bloodstream and are a considerable component of the general host response to tissue damage caused by cancer cells (Cortini et al., 2016; Avnet et al., 2017; Cortini et al., 2017). In another breast cancer metastatic model, MSC stimulate tumor extravasation and activation of the cancer cell receptor CXCR2 and the bone-secreted chemokine CXCL5 (Bersini et al., 2014). Chemokines and interleukins are also responsible for chemo-attraction of immune cells that, once recruited to the niche, become part of the tumor bulk and play a fundamental role in the tumor TME. Microfluidic platforms retain also the possibility to monitor immune cell migration and analyze their contribution to the formation of the metastatic niche through spatial compartmentalization (Gopalakrishnan et al., 2015; Boussommier-Calleja et al., 2016).

**Table 1:** 3D models for bone cancers.

3D model	Tumor setting	Relevance	References
3D collagen gel system containing osteoblast-like cells	Metastasis from endometrial, prostate and breast cancer	Prostate cancer cells produced morphological evidence of blastic reaction and evidence of local invasion	Sourla et al., 1996
3D hybrid hydrogel system composed of collagen and alginate	Invasive breast cancer Osteosarcoma	Human mammary fibroblast cells facilitated migration of breast cancer cells out of spheroids and into the surrounding matrix	Thakuri et al., 2018
3D spheroids in combination with 2D endothelial cells		Formation of tubule-like structures that mimic vessel sprouting and angiogenesis	Chaddad et al., 2017
Microfluidic niche-on-a-chip	Metastatic breast cancer	Formation of a self-assmebled vasculature network supported by MSC	Marturano-Kruik et al., 2018
Triculture system in microfluidics	Metastatic breast cancer	Extravasation and micrometastasis generation of breast cancer cells within a bone-like microenvironment	Bersini et al., 2014
Microfluidics bone-marrow-on-a-chip	Hematological diseases	Analysis of drug responses and toxicity	Torisawa et al., 2014
Microfluidics bone-on-a-chip	Metastatic breast cancer	Interaction between cancer cells and bone matrix that lead to tumor colonization	Hao et al., 2018
Bioreactors	Ewing sarcoma	Recreation of the bone niche that mimics native tumor properties	Marturano-Kruik et al., 2016
Bone scaffold	Ewing sarcoma	Analysis of cell cytotoxicity to respect to 2D	Fong et al., 2013
Spheroids	Osteosarcoma	Analysis of chemoresistance	Arai et al., 2013
Spheroids	Osteosarcoma	Analysis of cell cytotoxicity to doxorubicin	Baek et al., 2016a
Spheroids	Osteosarcoma	Analysis of cell cytotoxicity to cisplatin	Baek et al., 2016b
Spheroids	Osteosarcoma	Analysis of cell chemoresistance to oxidovanadium(IV)	Leon et al., 2016
Spheroids	Chondrosarcoma	Analysis of cell chemoresistance to doxorubicin and mafosfamide	Monderer et al., 2013
Spheroids	Chondrosarcoma	Analysis of cell cytotoxicity to respect to salinomycin	Perut et al., 2018
Microfluidics co-culture of tumor and MSC	Ewing sarcoma	Resistance of tumor cells to IGF-1R inhibitors due to the presence of MSC	Santoro et al., 2017


Microfluidics have been also used to successfully recreate the complex bone marrow TME (Torisawa et al., 2014). The microchip device included two different compartments: one for the specific growth of osteoblasts and one for medium change. Because osteoblastic tissues require long-term cultures, this design was proven successful as it allowed the formation of a thick mineralized osteoblastic tissue *in vitro* in a 1 month time period (Hao et al., 2018). The bone niche can be also recreated by using bone scaffolds (Marturano-Kruik et al., 2016). In ES, for example, cell growth rate is far slower *in vivo* than that observed *in vitro*, thereby more likely reproducing reliable growth conditions (Fong et al., 2013) and mimicking critical signaling cascades, such as the IGF-1R/PI3K7mTOR signaling pathway (Lamhamedi-Cherradi et al., 2014).

## Assaying Chemoresistance in 3D *In Vitro* Systems of Bone Cancers

Arai et al. (2013) found that spheroid cells displayed more chemoresistance to doxorubicin corresponding to higher IC50 values than conventional monolayer cells (Baek et al., 2016a) in more than 11 OS cell lines. Similarly, Baek et al. (2016b) confirmed that OS cells were more chemoresistant in 3D compared to 2D culture. Similar results were obtained also with cisplatin. Likewise, the use of MG-63 spheroids effectively predicted the cytotoxicity of oxidovanadium(IV) *in vivo* models (Leon et al., 2016). 3D CS cultures are resistant to doxorubicin and mafosfamide, when compared to standard monolayer cultures (Monderer et al., 2013). However, the use of 3D spheroids allowed to reveal that treatment with the ionophore salinomycin, previously uncharacterized for its effects on CS, significantly enhanced the cytotoxic effect to doxorubicin in 3D structures (Perut et al., 2018).

Finally, drug sensitivity of tumor cells might be strongly affected by microenvironmental factors that include the presence of MSC (Avnet et al., 2017; Senthebane et al., 2017), also when co-cultured with cancer cells in 3D structures. As an example, the 3D assembly of ES cells with MSC elicits ligand-mediated activation of the insulin-like growth factor-1 receptor (IGF-1R), thereby mediating resistance to IGF-1R inhibitors (Santoro et al., 2017).

## From Preclinical Models to Clinical Validation

The main aim of expanding the knowledge on culture systems is to be able to translate the molecular features of the 3D cell cultures of individual patients using them as a platform for drug screening and to identify biomarkers and new drug targets (Breslin and O’Driscoll, 2013). Findings using 3D models that more accurately reflect human sarcoma biology are likely to translate into improved clinical outcomes (Gao et al., 2017). As an example moving in this direction, Pauli and colleagues have made a great effort in describing a precision-medicine platform that integrates whole-exome sequencing with a living biobank that enables high-throughput drug screens on patient-derived tumor organoids. To date, 56 tumor-derived organoid cultures and 19 patient-derived xenograft models have been established from the 769 patients enrolled in an Institutional Review Board–approved clinical trial (Pauli et al., 2017). These types of approach have the potential not only to select the appropriate therapeutic option, but also to improve the knowledge on the molecular cues that lay at the basis of tumor development.

## Conclusion

Three dimensional models offer early promise in establishing robust preclinical platforms for the identification of crucial molecular pathways and for the assessment of clinical efficacy of novel drugs to inhibit cancer development and progression. Despite the perfect model currently does not exist and 3D approaches are characterized by weaknesses, these greatly expand the spectrum of cancer subtypes that might be considered for new drug screening and for the development of personalized medicine. In the field of bone cancers, rare and deadly diseases, this is of paramount importance to improve the clinical outcomes.

## Author Contributions

All authors listed have made a substantial, direct and intellectual contribution to the work, and approved it for publication.

## Conflict of Interest Statement

The authors declare that the research was conducted in the absence of any commercial or financial relationships that could be construed as a potential conflict of interest.
